# Comparison of nanoimaging and nanoflow based detection of extracellular vesicles at a single particle resolution

**DOI:** 10.1002/jex2.70016

**Published:** 2024-10-16

**Authors:** Shihan Xu, Zhengrong Zhang, Bridgette C. Melvin, Nibedita Basu Ray, Seiko Ikezu, Tsuneya Ikezu

**Affiliations:** ^1^ Department of Neuroscience Mayo Clinic Florida Jacksonville Florida USA; ^2^ Regenerative Science Graduate Program Mayo Clinic College of Medicine and Science Jacksonville Florida USA

**Keywords:** direct stochastic optical reconstruction microscopy, extracellular vesicle, machine learning, nanoflow cytometry, nanoimaging

## Abstract

The characterization of single extracellular vesicle (EV) has been an emerging tool for the early detection of various diseases despite there being challenges regarding how to interpret data with different protocols or instruments. In this work, standard EV particles were characterized for single CD9^+^, single CD81^+^ or double CD9^+^/CD81^+^ tetraspanin molecule positivity with two single EV analytic technologies in order to optimize their EV sample preparation after antibody labelling and analysis methods: NanoImager for direct stochastic optical reconstruction microscopy (dSTORM)‐based EV imaging and characterization, and Flow NanoAnalyzer for flow‐based EV quantification and characterization. False positives from antibody aggregates were found during dSTORM‐based NanoImager imaging. Analysis of particle radius with lognormal fittings of probability density histogram enabled the removal of antibody aggregates and corrected EV quantification. Furthermore, different machine learning models were trained to differentiate antibody aggregates from EV particles and correct EV quantification with increased double CD9^+^/CD81^+^ population. With Flow NanoAnalyzer, EV samples were prepared with different dilution or fractionation methods, which increased the detection rate of CD9^+^/CD81^+^ EV population. Comparing the EV phenotype percentages measured by two instruments, differences in double positive and single positive particles existed after percentage correction, which might be due to the different detection limit of each instrument. Our study reveals that the characterization of individual EVs for tetraspanin positivity varies between two platforms—the NanoImager and the Flow NanoAnalyzer—depending on the EV sample preparation methods used after antibody labelling. Additionally, we applied machine learning models to correct for false positive particles identified in imaging‐based results by fitting size distribution data.

## 1 INTRODUCTION

Extracellular vesicles (EVs) are membrane‐coated nanoparticles released from cells by exocytosis. EVs, ranging in size from 40 to 1000 nm, contain biological cargo such as lipids, proteins, DNA and RNA (Théry et al., 2002). EVs are found in most body fluids, playing key roles in biological processes such as coagulation, intercellular signalling, immune responses, cellular waste management (EL Andaloussi et al., 2013) and tumour metastasis (Costa‐Silva et al., 2015; Hoshino et al., 2015). EVs are highly heterogeneous in molecular composition (Poliakov et al., 2009; Yuana et al., 2013), and their surface proteins bear unique characteristics of their cells of origin (Larssen et al., 2017), such that specific subclasses of these vesicles reflect the pathology of specific tissues (Peinado et al., 2012) are thus a promising platform for tissue‐specific biomarkers and liquid biopsy (Santiago‐Dieppa et al., 2014; Yoshioka et al., 2014). EVs have the potential to detect molecular changes preceding clinical symptoms as prognostic markers (Cano et al., 2023).

To understand their function, it is critical to obtain detailed information about the EV surface proteins, such as copy number of each protein, their spatial distribution and the interactions between various types of proteins. For bulk sample analysis of EVs, conventional and digital ELISA, as well as western blot, were applied for protein quantification, which had its own disadvantages including requirement of large amounts of samples, extensive isolation process for EVs, low sensitivity and missing information for the heterogeneity of individual EVs. Small size and relatively low protein abundance in EVs makes it difficult to detect them by conventional flow cytometry (van der Pol et al., 2014). In recent years, novel technologies have been developed with increased sensitivity of molecular profiling of EVs, including multielectrode spectroscopy (Kilic et al., 2022), nano‐plasmonic sensors (Im et al., 2014) and nanopatterned microfluidic chips (Zhang et al., 2020, 2019) There are some new technologies focused on individual EVs as well, such as super resolution imaging (Lee et al., 2018) and nanoscale flow cytometry (Higginbotham et al., 2016).

Nano‐flow cytometry was developed to characterize EV particle concentration and their protein profiling (Chen et al., 2023; Dong et al., 2021; Wei et al., 2020; Yang et al., 2021), including using commercial instruments, for example, Amnis CellStream (Cytek) (von Lersner et al., 2024), a BD FACSAria III SORP cell sorter (Beckton Dickinson) (Ali Moussa et al., 2022) and Flow NanoAnalyzer (NanoFCM) (Kobayashi et al., 2024). For Flow NanoAnalyzer, it is capable of light scattering detection of single silica nanoparticles and EVs as small as 24 and 40 nm, respectively (Tian et al., 2018). The fluorescence detection limit was three Alexa Fluor 532 molecules (Zhu et al., 2014). The superior sensitivity of Flow NanoAnalyzer in the concurrent light scattering and fluorescence measurement of single EVs enables quantitatively profiling EV particles.

Direct stochastic optical reconstruction microscopy (dSTORM) bypasses the diffraction limit (∼250 nm) by analysing photoswitching events of individual molecules. During the STORM reconstruction process, individual molecules can be precisely localized by determining their position coordinates from the photons detected for each activation event (often as a Gaussian function). Furthermore, dSTORM was employed to visualize EVs in three‐dimensions and to localize molecule clusters such as the tetraspanins CD81 and CD9 on the surface of individual EVs and quantify subpopulations based on tetraspanin markers (McNamara et al., 2022). dSTORM‐based commercial platform, NanoImager, was applied for single EV imaging related with various diseases, such as Alzheimer's disease (You et al., 2023), preeclampsia (Gebara et al., 2022), SARS‐CoV‐2 (Singh et al., 2023) and diabetes (Guda et al., 2023).

In this current work, both Nano‐flow cytometry and NanoImager platforms were tested to characterize individual EVs from the same samples, with subsequent comparison of results between the two platforms. For the accurate quantification of EVs using NanoImager, size fitting methods and machine learning models were developed to remove false positive particles. For Flow NanoAnalyzer based study, different sample preparation methods after antibody labelling were performed for the comparison.

## 2 RESULTS

**FIGURE 1:**
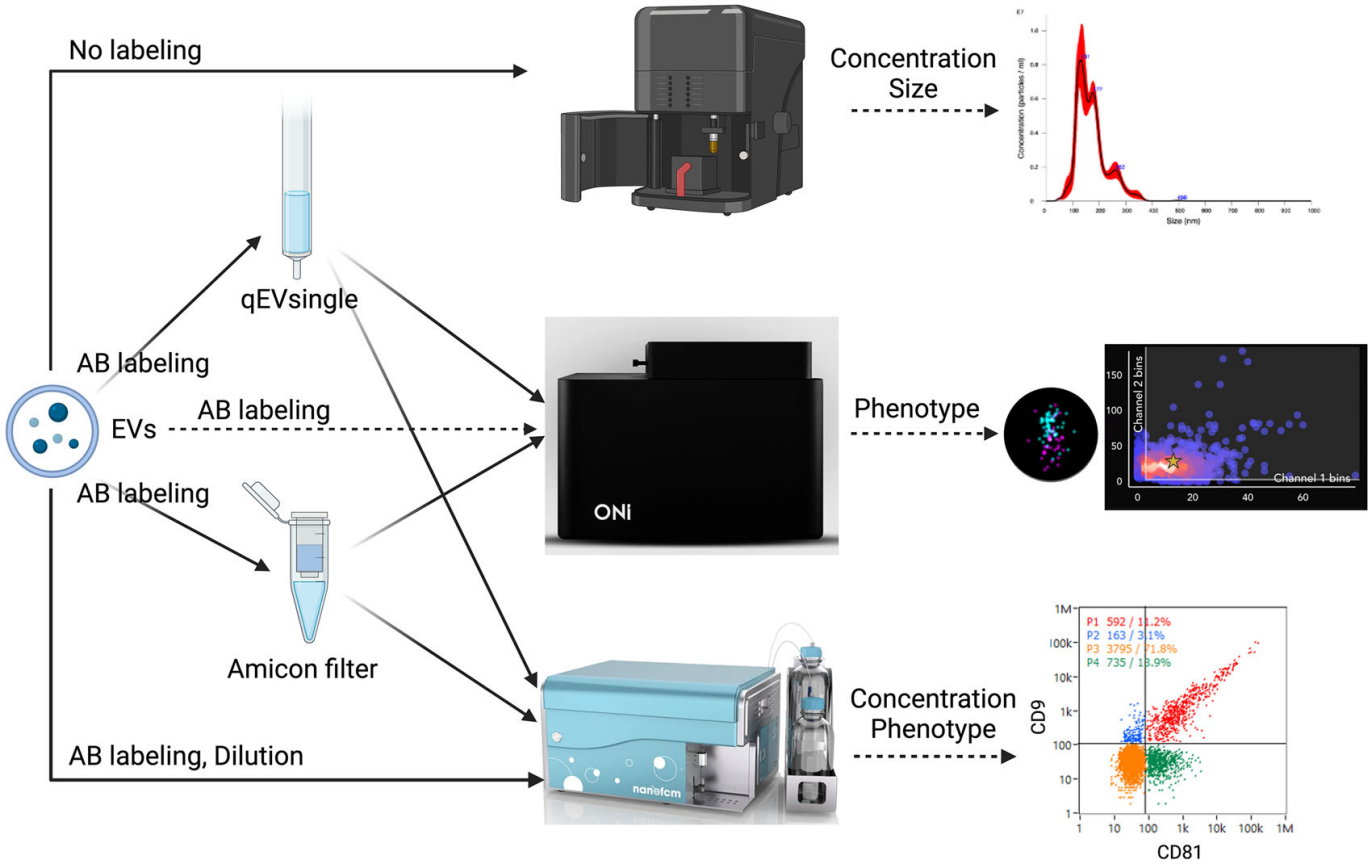
The schematic figure shows EVs were characterized by three instruments, including NTA, NanoImager and Flow NanoAnalyzer, regarding EV concentrations, sizes, and phenotypes. After EVs were labelled with fluorescent antibodies, two methods were chosen to remove free dyes, including Amicon filters and qEV single columns. NTA, nanoparticle tracking analysis.

First, nanoparticle tracking analysis (NTA), Flow NanoAnalyzer and NanoImager were used to characterize standard EV particles derived from the HCT116 human colorectal cell line (ONI company), including concentration and sizes (Figure 1 and Figures S1,S2). The mean size of EV particles measured by three instruments was 174 nm (NTA), 120 nm (NanoImager) and 84 nm (Flow NanoAnalyzer), respectively (Figure S1a–c).

CD9, CD63 and CD81 are known markers for EVs and play functional roles in EV biogenesis (Hurwitz et al., 2018; Pegtel & Gould, 2019). In this work, standard EVs were labelled with fluorophore‐conjugated antibodies against two tetraspanin markers (anti‐CD9‐A488 and anti‐CD81‐A647). All tested EVs were labelled with fluorescent antibodies against CD9 and CD81 in double‐filtered PBS (dfPBS) and the reference concentration of EVs in dfPBS was used for the measurement by Flow NanoAnalyzer. To reduce the signal from unbound fluorophore, EV samples were prepared via dilution method and were purified by either Amicon filter or purified by qEVsingle size exclusion column. Later they were tested by Flow NanoAnalyzer and NanoImager to evaluate the particle size and EV markers. To reduce EV loss, bovine serum albumin (BSA) was added to Amicon filters for pretreatment. The particle numbers of EVs before labelling and after labelling and fractionation with Amicon filters was recorded with Flow NanoAnalyzer, and the recovery rate was more than 90% (Figure S1e,f). Without using the BSA pretreatment for Amicon filters, the recovery rate was less than 30% (data not shown). After EV fractionation using qEVsingle column, the concentration of the EV sample dramatically dropped by approximately 100‐fold after fractionation (Figure S1d).

**FIGURE 2:**
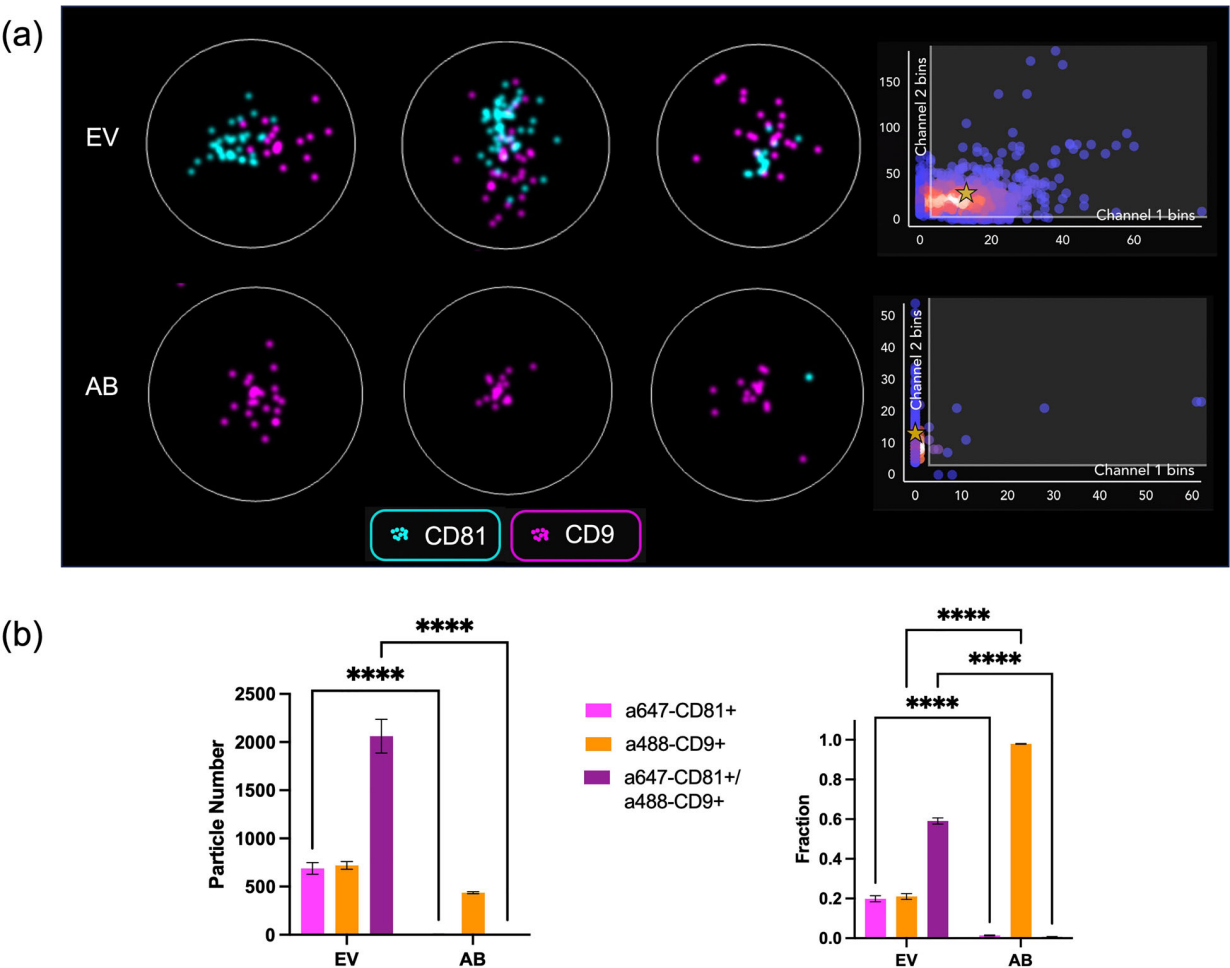
(a) EVs were labelled with two tetraspanin markers, including AF488 anti‐human CD9 and AF647 anti‐human CD81. EVs were captured and imaged as clusters under NanoImager, and further characterized, including single CD81^+^ (Cyan, Channel 1), and single CD9^+^ (Magenta, Channel 2) and CD9^+^/CD81^+^, EVs. In the scatter plot, the threshold was set as 3 for both channels. Control samples with only antibodies were tested following the same procedures. The white circle has a diameter of 300 nm. (b) The counts and fractions for each phenotype were analysed, and there were significant differences between labelled EV samples and control antibodies. Comparisons between EV and AB groups were conducted using two‐way ANOVA. Statistical significance was defined as *p* value < 0.05.

To capture EVs onto coverslips for dSTORM imaging, the coverslip surface was modified with (3‐Mercaptopropyl) trimethoxysilane (MPTS) at first, providing the sulfhydryl group (–SH) on the surface. Then Sulfo‐GMBS (N‐γ‐maleimidobutyryl‐oxysulfosuccinimide ester) (GMBS) was added to facilitate the reaction of the maleimide group with –SH. Amine groups from proteins (e.g., antibodies) can conjugate with NHS ester and bind onto the coverslips. The functionality of MPTS and GMBS to bind with such protein was confirmed by testing mouse‐IgG (Figure S3). To target mouse‐IgG, Alexa 488‐conjugated anti‐mouse IgG or Alexa 488‐conjugated anti‐rabbit IgG was added. To capture generic EVs, T‐cell immunoglobulin and mucin domain containing 4 (TIM‐4) was successfully modified on the surface of coverslips through phosphatidylserine reaction, and then labelled EVs were characterized with NanoImager at a single‐molecule resolution (Figure 2a).

With NanoImager, the average particle numbers of single CD81^+^, single CD9^+^ and CD9^+^/CD81^+^ EVs were 688 ± 61, 719 ± 40 and 2061 ± 175, respectively, while the fractions of single CD81^+^, single CD9^+^ and CD9^+^/CD81^+^ EVs were 0.20 ± 0.015, 0.21 ± 0.015 and 0.59 ± 0.016, respectively (Figure 2b, Table S1). Control samples of each antibody that did not have EVs were tested for non‐specific binding between the modified surface of the coverslip and antibody aggregates. Among all the fluorescent‐positive particles, the fractions of single CD81^+^ and single CD9^+^ particles were 0.014 ± 0.003 and 0.980 ± 0.002 (Figure 2b right), respectively, showing the high non‐specific binding of anti‐CD9 antibody onto the coverslip surface. On the other hand, the fraction of CD9^+^/CD81^+^ double positive particles were only 0.007 ± 0.002. Comparing the particle counts for double positive particles from both labelled EV samples and antibody control samples, the fraction of double positive particle from antibody control samples were very low, indicating that most double positive particles detected in the EV samples were from fluorescent antibody labelled EV particles, not from false positives caused by antibody aggregates.

**FIGURE 3:**
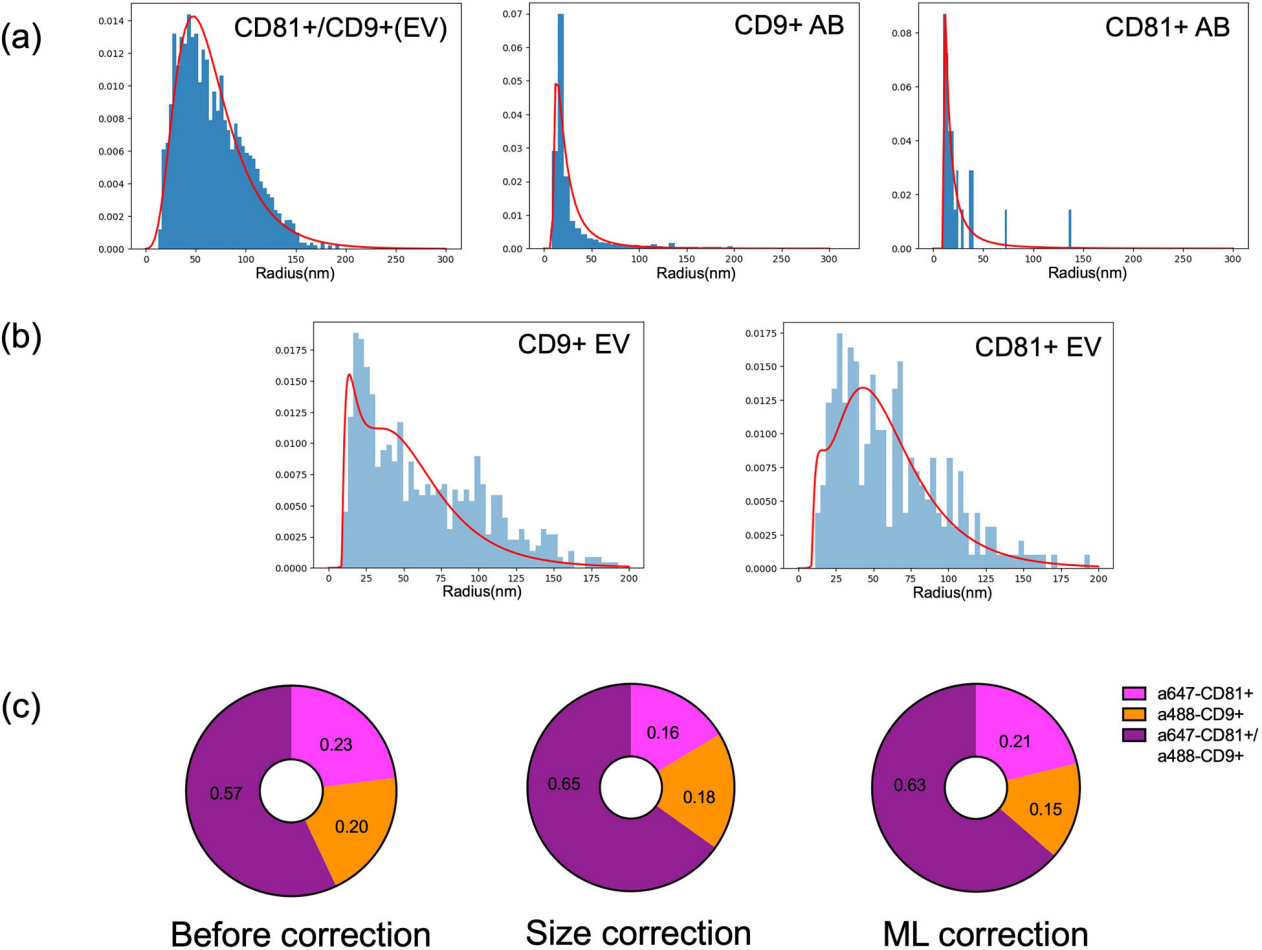
Normalized histograms showing probability density of radius of double positive particles (EVs) and antibody aggregates were fitted with lognormal functions. (a)–(b) Normalized histograms of the radius of single positive particles were fitted using 2 lognormal functions for antibody aggregates (blue) and CD9^+^/CD81^+^ double positive EV particles (green). *R* ^2^ values were 0.81 and 0.85 for the fitting of radius histograms of CD9^+^ EV and CD81^+^ EVs (Red), respectively. (c) By removing the antibody aggregates in single CD9^+^ EV and CD81^+^ EVs, percentages of each phenotype were recalculated, with size correction or ML correction. ML, machine learning.

To better differentiate the antibody aggregates from EV particles, radius of gyration, which is a feature in CODI NanoImager software, and it was chosen to characterize EVs and antibody aggregates. Normalized histograms with probability density for the radius of CD9^+^/CD81^+^ double positive particles (EV) and antibody aggregates were fitted with lognormal functions. The median value of ‘approximate radius’ was ∼60 nm for double positive EV particles. On the contrary, the median value of ‘approximate radius’ of antibody (AB) aggregates (CD9 or CD81) was only ∼20 nm (Figure 3a).

To remove antibody aggregates from single positive particles, the histogram for approximate radius of single positive particles was fitted using 2 lognormal functions for CD9^+^/CD81^+^ double positive EV particles and antibody aggregates and the areas under those two curves and their individual percentages were calculated (Figure 3b). By removing the estimated number of AB aggregates in single positive particles, percentages of each phenotype were corrected. For CD9^+^ single positive particles, percentage of EV and AB aggregates was accounted for 66.6% and 33.4%, respectively. For CD81^+^ single positive particles, percentage of EV and antibody accounted for 84.4% and 15.6%, respectively (Figure 3b). Before using correction with lognormal fitting, fractions for double positive, single CD9^+^ and CD81^+^ EV fractions were 0.57, 0.20 and 0.23. After size correction, fractions for single CD9^+^ and single CD81^+^ EVs were 0.18 and 0.16, respectively, and the double positive EV fraction increased to 0.65 (Figure 3c).

**FIGURE 4:**
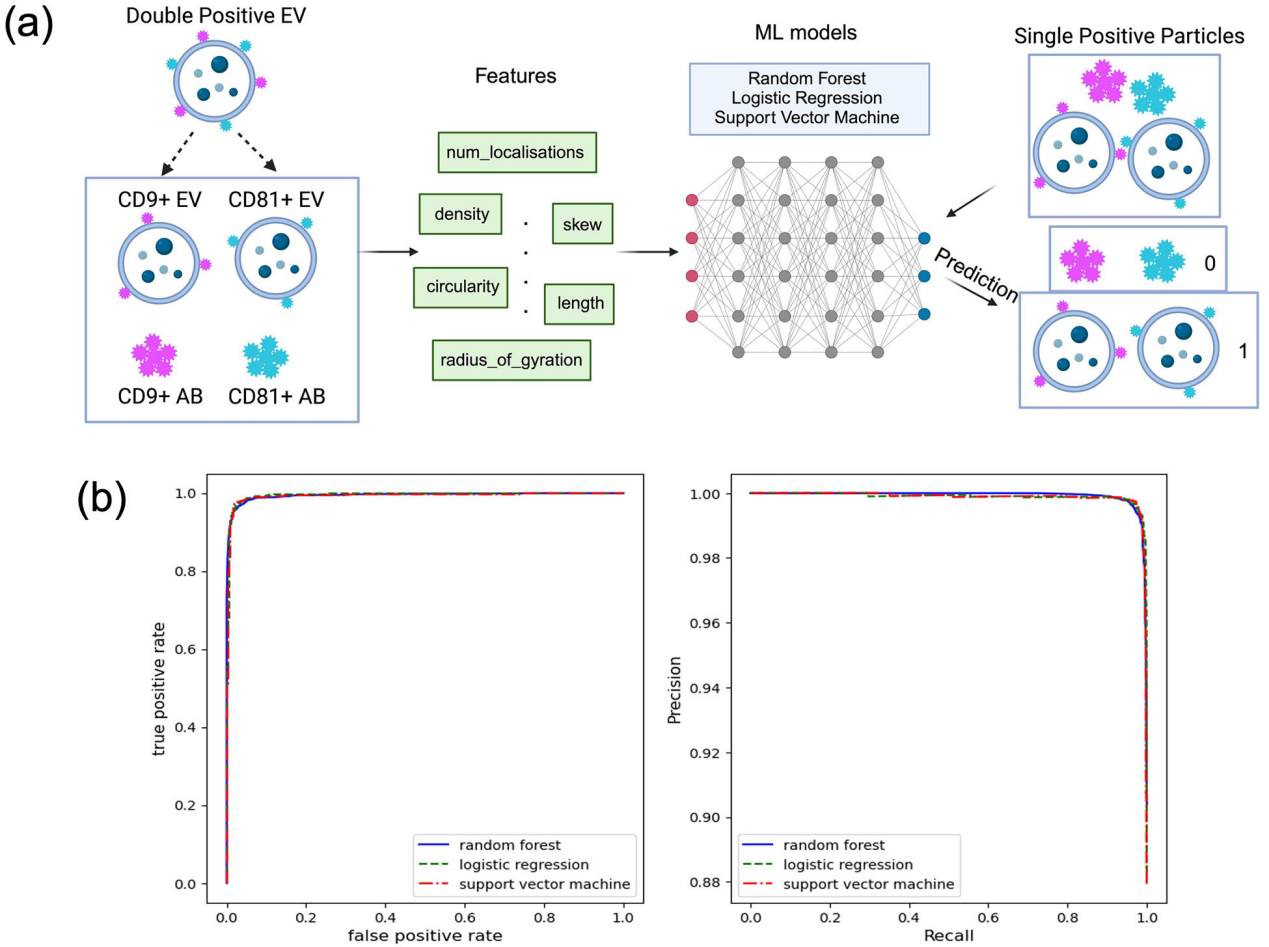
Application of machine learning. (a) Three machine learning models including random forest, logistic regression, and support vector machine, were applied for classification between antibody aggregates (‘0′) and EV particles (‘1′), based on various features (e.g., circularity, density and number of localizations). (b) Left: AUC‐ROC curves for three models, which provide graphical representations of the performance of models at different thresholds. Right: the trade‐off between precision and recall scores at different thresholds.

Different machine learning models (random forest, logistic regression, support vector machine) were applied to classify antibody aggregates and EV particles from single positive particles, based on CODI's features from NanoImager software (Figure 4a). To compare the performance of these three models, their accuracy, precision, recall, and AUC‐ROC scores were calculated with four technical replicate datasets using same EV samples (Table S2). The AUC‐ROC curves and relations between precision and recall scores show the high performance to detect EV particles using three machine learning models (Figure 4b). Overall, random forest model has the best AUC‐ROC score and performance among three models. There were significant differences depicted in features between two classes, AB group and EVs, including density, convex hull area, discretized area, radius of gyration, length, distance birth and Channel 1 binned counts (Figure S5). SHapley Additive exPlanations (SHAP) analysis was applied to visualize the importance of different features, highlighting the contribution of each feature on prediction (Figure S6).

Based on the classification, single positive particles, which were predicted as antibody aggregates, were removed, and the counts and percentages of CD9^+^/CD81^+^ double positive and single positive particles were thus corrected. The fractions for single CD9^+^ and single CD81^+^ EVs were 0.15 and 0.21, respectively. The double positive fraction increased to 0.63 (Figure 3c). After correction with both size fitting and machine learning for classification (random forest), the fraction of double positive EVs increased, despite the varied values of each phenotype.

### 2.1 Comparison with Flow NanoAnalyzer

The same EV samples were tested with Flow NanoAnalyzer for parallel comparison with NanoImager. To decrease the baseline of fluorescent intensity coming from free antibodies, the sample was diluted with different factors ranging from 50 to 400 folds to ensure no representative peaks from EV particles were masked under high baseline from free fluorescent antibodies or unconjugated free dye. As the dilution factor increased, the baselines decreased to a certain value. The percentage of CD9^+^/CD81^+^ double positive EVs increased, until it reached its saturation point at 5% (Figure S7).

**FIGURE 5:**
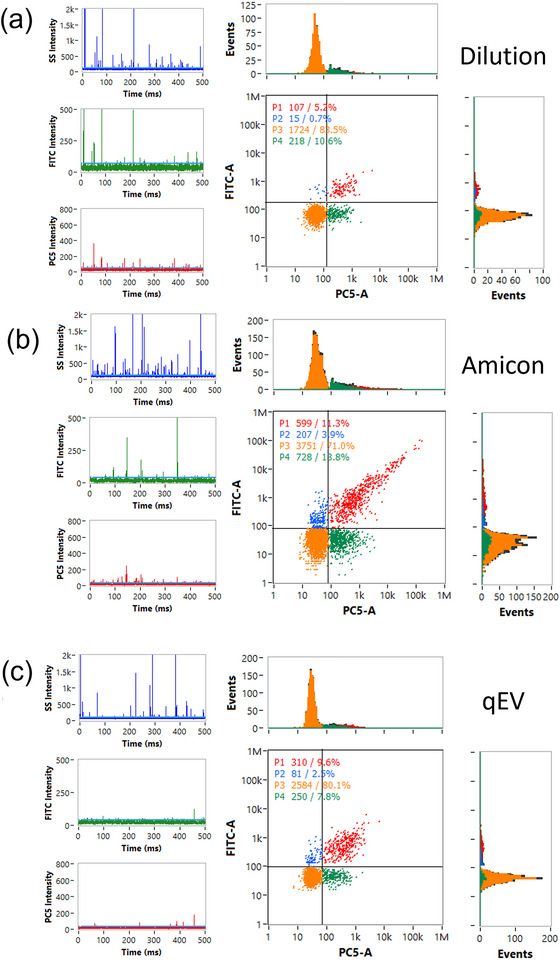
Flow NanoAnalyzer‐based characterization of EVs. Phenotypes of EV samples were tested after different approaches, including (a) dilution, (b) Amicon filters and (c) qEV column. 500 ms of flow intensity traces for detecting EV particles were recorded in three channels, including size‐scattering intensity (Blue), fluorescent intensity from AF488‐CD9 (Green) and AF647‐CD81 (Red), respectively. The histograms of the fluorescent intensity of single CD9 or C81 channel, and scatter plots were shown. (d) After removing double negative particles, the percentages of each phenotype were recalculated.

After diluting by 400 times, the percentages for CD9^+^/CD81^+^ double positive, single CD81^+^, and single CD9^+^ particles were 5.2%, 10.6% and 0.7% (Figure 5a), respectively. To remove free antibodies or unconjugated free dye from labelled EV samples, two methods, including Amicon filters and qEVsingle columns were applied. With pretreated Amicon filters, unconjugated free dye can be removed, and baseline value for detection decreased. The percentages for CD9^+^/CD81^+^ double positive, single CD81^+^, and single CD9^+^ particles were 11.2%, 13.9% and 3.1%, showing more positive particles detected with decreased baselines (Figure 5b). When using qEVsingle columns for EV samples, the percentage of CD9^+^/CD81^+^ double positive EVs was 9.6% (Figure 5c). After removing double negative particles from the results measured with Flow NanoAnalyzer, the percentages of each phenotype were recalculated for comparison with results measured from NanoImager (Figure 5d). EVs without any antibody labelling and mixed antibodies without EVs were tested as control samples (Figure S8).

Comparing these three sample preparation methods after antibody labelling, dilution was the most flexible method for adjusting the EV concentration and lowering background signal (Table 1). Initial concentrations of EV particles and fluorescent antibodies could be titrated. For Amicon filters and qEVsingle columns, similar double positive percentages were achieved, when EVs were fully labelled with fluorescent antibodies.

**TABLE 1:** The summary of different sample preparation methods after antibody labelling.

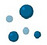	Dilution	UF	SEC
**Detection size**	∼85 nm	∼69 nm	∼68 nm
**Time**	∼2 min	∼30 min	∼60 min
**Pros**	Fast, easy Suitable for NanoImaging	Remove free dyes, low background signal for Flow NanoAnalyzer	Removing free dyes/antibodies/large particles, low background signal for Flow NanoAnalyzer)
**Cons**	High background signal (Flow NanoAnalyzer)	Time consuming, No improvement for NanoImager	Time consuming, not compatible with NanoImager

## 3 DISCUSSION

Our study demonstrates the florescence‐based characterization of tetraspanin positivity in individual EV at a single vesicle level using NanoImager and Flow NanoAnalyzer. Our results indicate that there is a potential masking in the detection of single molecules on EVs due to the antibody aggregation. With NanoImager, the percentage of CD9^+^/CD81^+^ double positive EVs increased after the removal of false positives created by each antibody, using lognormal fitting for radius distributions or machine learning models. With Flow NanoAnalyzer‐based fluorescence detection on individual EV particles, Amicon filters and qEV columns were applied to remove background fluorescence signals caused by unbound fluorescent dye and subsequently reduce the baseline fluorescent threshold in case a higher baseline masked out weak fluorescent signals from EV particles. Different antibody concentrations and dilution factors were also tested to achieve optimal conditions for baselines from fluorescent antibodies and EV particle concentration. For Flow NanoAnalyzer, after discarding the double negative particles, the percentages of double positive particles were recalculated to 48% (Amicon filter). The percentage of double positive EVs measured with NanoImager (63% with machine learning models) was much higher than that measured with Flow NanoAnalyzer (48%), implying NanoImager has much higher sensitivity with single molecule resolution than Flow NanoAnalyzer. Overall, NanoImager imaging has a higher sensitivity and a higher resolution for single‐molecule detection. Flow NanoAnalyzer, on the other hand, has a superior throughput.

Although the qEV columns caused significant EV dilution and loss, we were still able to characterize the concentration and phenotypes of EVs by Flow NanoAnalyzer. With NanoImager, however, only a few EVs were captured after qEV columns (Data not shown). This could be due to the materials in the column, affecting the binding of qEV‐purified EVs onto the coverslip. The usage of Amicon filters did not change the results of EV phenotypes with NanoImager, indicating that most of the antibody aggregates could not be removed with Amicon filters.

When profiling EV particles by Flow NanoAnalyzer, as shown in Figure S1, original EV size was 84.7 ± 18.2 nm, compared to 68.1 ± 17.7 nm after qEV Single column isolation. The size was 81.1 ± 18.2 nm after Amicon filteration once, and 68.8 ± 16.5 nm after Amicon filtration twice. One explanation is that larger particles are not EVs but contaminated particles, which were removed after qEV or Amicon based enrichment of EVs.

Comparing the concentration of standard EVs with NTA and Flow NanoAnalyzer, the concentration of EVs diluted in dfPBS was much higher when tested out by NTA compared to Flow NanoAnalyzer. In contrast, when EVs were diluted in double‐filtered water, the concentration measured by these two instruments were similar (Figure S2). This difference could be due to dfPBS, which may cause reduction in the flow speed of EV samples when tested by Flow NanoAnalyzer, leading to lower concentration output. Based on NTA results, there was no significant increase of EV particle sizes after dilution in water. Finally, single molecule detection is constrained by the detection limits of each instrument as the percentages of EV phenotypes varied between these two instruments.

## 4 CONCLUSION

In conclusion, we compared single extracellular vesicle (EV) characterizations for tetraspanin positivity using two platforms, the NanoImager and the Flow NanoAnalyzer, across various EV sample preparation methods after antibody labelling. For dSTORM imaging results, we corrected the phenotype percentages using size distribution fitting and machine learning models. Following these corrections, the NanoImager detected a significantly higher percentage of double‐positive EVs compared to the Flow NanoAnalyzer, suggesting that the NanoImager offers superior sensitivity. Additionally, different EV sample preparation methods resulted in varying phenotype percentages with the Flow NanoAnalyzer, highlighting the need for optimization and standardization in sample preparation to ensure consistent results when analysing EVs at the single‐particle level.

## 5 MATERIALS AND METHODS

### 5.1 Materials

Coverslips (22 mm * 48/22 mm * 22 mm, 1.5H, Thorlabs) were soaked in Contrad 70 (5%, v/v in Milli‐Q water, Decon Labs, 1002) for cleaning. For functional modification of coverslips, the following reagents were used: (3‐Mercaptopropyl) trimethoxysilane (MPTS, Sigma‐Aldrich, 175617), Sulfo‐GMBS (N‐γ‐maleimidobutyryl‐oxysulfosuccinimide ester) (GMBS, Thermo Fisher Scientific, 22324), Human TIM4/Human Fc Chimera, recombinant (TIM4, Fujifilm, 081–10261), mouse‐IgG (Santa Cruz Biotechnology, sc‐2025, Lot #F1819), Alexa 488 (AF488) goat anti‐mouse IgG (Invitrogen, 2306579), AF488 goat anti‐rabbit IgG (Invitrogen, Lot #25211557).

### 5.2 EV sample labelling

Standard EVs were supplied as part of the ONI Application kit: EV profiler 1, which comprised of 30 µg of lyophilized EVs derived from various biological materials, including cell‐conditioned media, human plasma, serum, and urine from pools of certified healthy donors. These exosomes/small EVs are purified using a combination of tangential flow filtration and size exclusion chromatography. The lyophilized EVs were reconstituted in 30 µL of Milli‐Q water. EVs (2.5 µL of 2*10^10^/mL, measured by Flow NanoAnalyzer) and were incubated with 1 µL of Anti‐CD9‐AF488, 1 µL of Anti‐CD81‐AF647 and 3.5 µL of B1 (ONI, Application kit: EV profiler 1) at 4°C overnight. To remove free antibodies from EV samples, Amicon Ultra Centrifugal filter (100 kDa MWCO, Amicon Ultra, Sigma‐Aldrich, UFC910008) and qEVsingle columns (70 nm, IZON, ISC‐70) were used for EV fractionation. EVs without any antibody labelling and mixed antibodies without EVs were tested as control samples.

### 5.3 Coverslip modification

Before any modification, coverslips were washed with water and ethanol three times. Ten microlitre of MPTS (4% v/v in 100% ethanol) was added to the surface of the coverslips and incubated for 0.5 h at room temperature. The coverslips were then washed with ethanol three times, and allowed to dry completely. Ten microlitre of GMBS (10 µM in PBS) was then added on the surface of the coverslips, incubated for 0.5 h, and then the coverslip was washed with dfPBS for three times. Ten microlitre of TIM4 (100 µg/mL, Fujifilm) was added onto the same spot, incubating for 0.5 h. After washing the coverslips with dfPBS, 1 µL of C1 buffer (ONI company kit) was added to the prelabelled EV sample, and then the EV sample was added onto the modified coverslip. After 1 h, the coverslip was washed with dfPBS three times. Ten microlitre of 4% paraformaldehyde was added on the coverslip for fixation and incubated for 15 min, then washed with dfPBS three times. Lastly, 10 µL of fresh imaging buffer was added on each spot and sealed with small coverslips before imaging.

To test the functionality of modified coverslips, 10 µL of mouse IgG (100 µg/mL) was added after the MPTS and GMBS modification. Then 10 µL of AF488 goat anti‐mouse or Alexa 488 goat anti‐rabbit IgG (100 µg/mL) was added to target mouse‐IgG.

### 5.4 Sample processing after antibody labelling

There were two methods to remove free dyes from EV samples after antibody labelling, including Amicon filters and qEV single size exclusion chromatography (SEC). When using Amicon filters, EV loss was found, and the percentage of loss also depended on the initial feeding concentrations of EV particles. To reduce particle loss, Amicon filters were pre‐treated with 0.1% BSA in dfPBS, then centrifuged at 5000 × *g* for 5 min at 4°C (Centrifuge 5424 R, Eppendorf). The remaining BSA was discarded, and the filter membrane was washed twice with dfPBS. Half mL of each EV sample was loaded into an Amicon Ultra filter and centrifuged at a speed of 10,000 rcf for 5 min to remove unbound antibodies. The remaining solution on the filter was collected as the filtered sample.

The qEVsingle column was used for EV sample fractionation. EV sample in 0.15 mL was loaded on the top of the column after column washing, then 0.7 mL dfPBS was added for elution. These eluates were discarded as flow through. Another 2 mL of dfPBS was added to elute EVs, and five fractions of eluate were collected (0.17 mL for each fraction, and the leftover volume was discarded). These fractions were tested by Flow NanoAnalyzer.

### 5.5 Super‐resolution microscopy

Super‐resolution images of Evs were acquired using dSTORM [Nanoimager S, Oxford Nanoimaging (ONI), UK] equipped with a 100×, 1.4NA oil immersion objective. The temperature was set at 31°C and the illumination angle for TIRF imaging was 53.5°. EV samples were immobilized on TIM4‐modified coverslips and stained with tetraspanin antibodies as previously described. Before capturing images, 100 nm Tetraspec beads (Thermo Fisher Scientific) were counted in two fluorescent channels for colocalization calibration as recommended in MIFlowCyt‐EV guideline (Lee et al., 2008). AF488‐anti‐human CD9 and AF647 anti‐human CD81 were illuminated with 488 and 637 nm lasers, respectively. One thousand five hundred frames of images were recorded for each channel, and the total number of frames for the two channels was 3000. The exposure time for one frame was 30 ms. Co‐localization analysis of two different molecules on single EVs was proceeded with the CODI online analysis platform (https://alto.codi.bio/). First, drift correction was performed for 3000 frames. Temporal grouping for quantification was set with frame gap as 2 and maximum distance as 30 nm. For the maximum distance, the larger the distance, the sparser the localization molecules were visualized for individual EV particles. Settings were adjusted from 20 to 60 nm (Figure S4). The optimal setting would depend on the size of EVs, fluorophores and the occurrence of molecules being detected. The setting of 30 nm was chosen as the optimal value for analysing dSTORM images (Golfetto et al., 2018). Localization clusters with more than 15 individual molecules were classified as EV clusters. For dSTORM imaging of EV samples, unbound dyes were not necessary to be removed, since they can be filtered out by the ML algorithms. EV clusters with Skew ranging from 1 to 2.9 and Circ ranging from 0.43 to 1 were selected for further analysis. An object was considered positive as a single EV particle when more than 3 individual molecules were detected in the same channel within a radius of 150 nm around the cluster's centre. Five fields of view were acquired from each sample for statistically analysis. False positive particles detected from bare coverslips and coverslips with modifications (MPTS/GMBS/TIM4) were less than 30 in each image and those too were filtered through the data analysis.

### 5.6 Flow NanoAnalyzer

Following the same EV sample labelling protocol, the concentrations of antibodies were optimized to achieve the saturated labelling condition for each antigen. Before testing samples, two calibration beads from the kit (NanoFCM) were tested, including QC beads for concentration calibration (fluorescence intensity detection) and size beads for size calibration (side‐scattering intensity detection, SSC) as previously recommended (Tian et al., 2020; Welsh et al., 2023).When testing EV samples, particles were detected based on SSC intensity, that only particles whose SSC intensity was higher than the default threshold value (automatically set based on its baseline and standard deviation value) were counted. light‐scattering sizing was calculated based on the SSC intensity from EV particles by referring to the calibration curve from size beads. For fluorescent detection of individual EV particles targeted by dye‐tagged antibodies, samples were illuminated with 488 and 647 nm lasers. Flow intensity traces of EV particles labelled with Anti‐CD9‐AF488 and Anti‐CD81‐AF647 were recorded in two channels. In Flow NanoAnalyzer, the bandpass filters installed were 580/40 and 710/40 nm.

For samples labelled with antibodies, different dilution factors were tested to find the optimal labelling conditions. The particle numbers from dfPBS buffer ranged from 200 to 400 particles per min. To minimize the effect of false positive particles from dfPBS buffer, the minimum particle number measured in each sample was set at 1000.

### 5.7 NTA

The size distribution and concentration of the samples was measured using the NanoSight NS300 instrument (Malvern, Worcestshire, UK). The NS300 utilizes NTA technology to visualize and track individual nanoparticles (include sample ID) based on their Brownian motion. The instrument was calibrated using polystyrene latex microspheres of known sizes (provided by the manufacturer) according to the manufacturer's guidelines. This calibration ensured accurate size measurements during the subsequent sample analysis. For each sample, video recordings were captured for four times, each time with a duration of 30 s and recorded at 25 frames per second. The recorded videos were analysed using the NanoSight NTA software (provided by the manufacturer). A size distribution histogram was generated based on the Brownian motion of the particles in each sample. From this analysis, the mean size, size distribution width, and concentration of particles in each sample were extracted. 

### 5.8 Size distribution fitting

From the output of CODI platform, the feature called ‘radius of gyration’ was chosen to characterize size difference between EV particles and antibody aggregates. Histograms of thousands of particles were plotted with 50 nm bin width, for double positive EVs and antibody aggregates. Furthermore, these two histograms were fitted using log‐normal function curves. Then, histograms of single positive particles (CD9^+^ or CD81^+^) were fitted by two log‐normal curves for EVs (double positive) and antibody aggregations (Figure 3). Weights for 2 lognormal curves were calculated. The numbers of single positive EVs were corrected after removing antibody aggregates. Python codes for size distribution fitting were uploaded on Github (https://github.com/ven000/lognormal‐fitting).

### 5.9 Machine learning models

Different machine learning models were applied for classification, including random forest, logistic regression, and support vector machine. Two classes including ‘double positive EVs’ and ‘antibody aggregates’ were labelled as ‘1′ and ‘0′, respectively. Distinctive features for these two classes from the output of super‐resolution images were applied to train three models, including radius of gyration, num_localisations, convex_hull_area, distance_birth, skew, discretised_area, distance_depth, circularity, density, length, Channel 1 binned counts, Channel 2 binned counts.

Double positive particles were divided into two entries (two single positive particles) for prediction of single positive particles. Since the numbers of antibody aggregates and double positive EVs varied significantly, the weight for the training data and test data was set to be ‘balanced’. Python codes for machine learning models were uploaded on Github (https://github.com/ven000/ml‐models).

Statistical analysis for Figure 2 and Figure S2.

## AUTHOR CONTRIBUTIONS

**Shihan Xu**: Data curation (lead); formal analysis (lead); investigation (lead); methodology (lead); software (lead); validation (lead); visualization (lead); writing—original draft (lead); writing—review and editing (lead). **Zhengrong Zhang**: Data curation (supporting); investigation (supporting); methodology (supporting); writing—review and editing (equal). **Bridgette C. Melvin**: Data curation (supporting); formal analysis (equal); investigation (supporting); methodology (supporting); project administration (supporting); validation (supporting); visualization (equal); writing—review and editing (equal). **Nibedita Basu Ray**: Data curation (supporting); formal analysis (supporting); investigation (supporting); methodology (supporting); validation (supporting); writing—review and editing (supporting). **Seiko Ikezu**: Funding acquisition (equal); project administration (supporting); resources (equal); supervision (equal); writing—original draft (equal); writing—review and editing (lead). **Tsuneya Ikezu**: Conceptualization (lead); funding acquisition (lead); project administration (lead); resources (lead); supervision (lead); writing—original draft (supporting); writing—review and editing (equal).

## CONFLICT OF INTEREST STATEMENT

The authors declare no conflicts of interest.

## CONSENT FOR PUBLICATION

All involved parties consented to publication of this work.

## REFERENCES

## Supporting information



Supporting Information

## Data Availability

The data produced and analysed in this study are all available herein. The datasets used for analysis are available from the corresponding author upon reasonable request. The raw data files of flow experiments were uploaded onto the GitHub (https://github.com/ven000/mayo).

## References

[jex270016-bib-0001] Ali Moussa, H. Y. , Manaph, N. , Ali, G. , Maacha, S. , Shin, K. C. , Ltaief, S. M. , Gupta, V. , Tong, Y. , Ponraj, J. , Salloum‐Asfar, S. , Mansour, S. , Al‐Shaban, F. A. , Kim, H. G. , Stanton, L. W. , Grivel, J. C. , Abdulla, S. A. , Al‐Shammari, A. R. , & Park, Y. (2022). Single extracellular vesicle analysis using flow cytometry for neurological disorder biomarkers. Frontiers in Integrative Neuroscience, 16, 879832. 35655952 10.3389/fnint.2022.879832 PMC9152125

[jex270016-bib-0002] Cano, A. , Esteban‐de‐Antonio, E. , Bernuz, M. , Puerta, R. , García‐González, P. , de Rojas, I. , Olivé, C. , Pérez‐Cordón, A. , Montrreal, L. , Núñez‐Llaves, R. , Sotolongo‐Grau, Ó. , Alarcón‐Martín, E. , Valero, S. , Alegret, M. , Martín, E. , Martino‐Adami, P. V. , Ettcheto, M. , Camins, A. , Vivas, A. , … Ruíz, A. (2023). Plasma extracellular vesicles reveal early molecular differences in amyloid positive patients with early‐onset mild cognitive impairment. Journal of Nanobiotechnology, 21(1), 1–19. 36788617 10.1186/s12951-023-01793-7 PMC9930227

[jex270016-bib-0003] Chen, C. , Cai, N. , Niu, Q. , Tian, Y. , Hu, Y. , & Yan, X. (2023). Quantitative assessment of lipophilic membrane dye‐based labelling of extracellular vesicles by nano‐flow cytometry. Journal of Extracellular Vesicles, 12(8), e12351. 37525378 10.1002/jev2.12351 PMC10390660

[jex270016-bib-0004] Costa‐Silva, B. , Aiello, N. M. , Ocean, A. J. , Singh, S. , Zhang, H. , Thakur, B. K. , Becker, A. , Hoshino, A. , Mark, M. T. , Molina, H. , Xiang, J. , Zhang, T. , Theilen, T. M. , García‐Santos, G. , Williams, C. , Ararso, Y. , Huang, Y. , Rodrigues, G. , Shen, T. L. , … Lyden, D. (2015). Pancreatic cancer exosomes initiate pre‐metastatic niche formation in the liver. Nature Cell Biology, 17(6), 816–826. 25985394 10.1038/ncb3169 PMC5769922

[jex270016-bib-0005] Dong, L. , Huang, C. Y. , Johnson, E. J. , Yang, L. , Zieren, R. C. , Horie, K. , Kim, C. J. , Warren, S. , Amend, S. R. , Xue, W. , & Pienta, K. J. (2021). High‐throughput simultaneous mRNA profiling using nCounter technology demonstrates that extracellular vesicles contain different mRNA transcripts than their parental prostate cancer cells. Analytical Chemistry, 93(8), 3717–3725. 33596381 10.1021/acs.analchem.0c03185 PMC7944479

[jex270016-bib-0006] EL Andaloussi, S. , Mäger, I. , Breakefield, X. O. , & Wood, M. J. (2013). Extracellular vesicles: Biology and emerging therapeutic opportunities. Nature Reviews Drug Discovery, 12(5), 347–357. 23584393 10.1038/nrd3978

[jex270016-bib-0007] Gebara, N. , Scheel, J. , Skovronova, R. , Grange, C. , Marozio, L. , Gupta, S. , Giorgione, V. , Caicci, F. , Benedetto, C. , Khalil, A. , & Bussolati, B. (2022). Single extracellular vesicle analysis in human amniotic fluid shows evidence of phenotype alterations in preeclampsia. Journal of Extracellular Vesicles, 11(5), e12217. 35582873 10.1002/jev2.12217 PMC9115584

[jex270016-bib-0008] Golfetto, O. , Wakefield, D. L. , Cacao, E. E. , Avery, K. N. , Kenyon, V. , Jorand, R. , Tobin, S. J. , Biswas, S. , Gutierrez, J. , Clinton, R. , Ma, Y. , Horne, D. A. , Williams, J. C. , & Jovanović‐Talisman, T. (2018). A platform to enhance quantitative single molecule localization microscopy. Journal of the American Chemical Society, 140(40), 12785–12797. 30256630 10.1021/jacs.8b04939 PMC6187371

[jex270016-bib-0009] Guda, P. R. , Sharma, A. , Anthony, A. J. , ElMasry, M. S. , Couse, A. D. , Ghatak, P. D. , Das, A. , Timsina, L. , Trinidad, J. C. , Roy, S. , Clemmer, D. E. , Sen, C. K. , & Ghatak, S. (2023). Nanoscopic and functional characterization of keratinocyte‐originating exosomes in the wound fluid of non‐diabetic and diabetic chronic wound patients. Nano Today, 52, 101954. 38282661 10.1016/j.nantod.2023.101954 PMC10810552

[jex270016-bib-0010] Higginbotham, J. N. , Zhang, Q. , Jeppesen, D. K. , Scott, A. M. , Manning, H. C. , Ochieng, J. , Franklin, J. L. , & Coffey, R. J. (2016). Identification and characterization of EGF receptor in individual exosomes by fluorescence‐activated vesicle sorting. Journal of Extracellular Vesicles, 5(1), 29254. 27345057 10.3402/jev.v5.29254 PMC4921784

[jex270016-bib-0011] Hoshino, A. , Costa‐Silva, B. , Shen, T. L. , Rodrigues, G. , Hashimoto, A. , Tesic Mark, M. , Molina, H. , Kohsaka, S. , Di Giannatale, A. , Ceder, S. , Singh, S. , Williams, C. , Soplop, N. , Uryu, K. , Pharmer, L. , King, T. , Bojmar, L. , Davies, A. E. , Ararso, Y. , … Lyden, D. (2015). Tumour exosome integrins determine organotropic metastasis. Nature, 527(7578), 329–335. 26524530 10.1038/nature15756 PMC4788391

[jex270016-bib-0012] Hurwitz, S. N. , Cheerathodi, M. R. , Nkosi, D. , York, S. B. , & Meckes, D. G. Jr. (2018). Tetraspanin CD63 Bridges autophagic and endosomal processes to regulate exosomal secretion and intracellular signaling of Epstein‐Barr virus LMP1. Journal of Virology, 92(5), e01969‐e01917. 10.1128/JVI.01969-17 29212935 PMC5809724

[jex270016-bib-0013] Im, H. , Shao, H. , Park, Y. I. , Peterson, V. M. , Castro, C. M. , Weissleder, R. , & Lee, H. (2014). Label‐free detection and molecular profiling of exosomes with a nano‐plasmonic sensor. Nature Biotechnology, 32(5), 490–495. 10.1038/nbt.2886 PMC4356947 24752081

[jex270016-bib-0014] Kilic, T. , Cho, Y. K. , Jeong, N. , Shin, I. S. , Carter, B. S. , Balaj, L. , Weissleder, R. , & Lee, H. (2022). Multielectrode spectroscopy enables rapid and sensitive molecular profiling of extracellular vesicles. ACS Central Science, 8(1), 110–117. 35111901 10.1021/acscentsci.1c01193 PMC8802188

[jex270016-bib-0015] Kobayashi, H. , Shiba, T. , Yoshida, T. , Bolidong, D. , Kato, K. , Sato, Y. , Mochizuki, M. , Seto, T. , Kawashiri, S. , & Hanayama, R. (2024). Precise analysis of single small extracellular vesicles using flow cytometry. Scientific Reports, 14(1), 7465. 38553534 10.1038/s41598-024-57974-3 PMC10980769

[jex270016-bib-0016] Larssen, P. , Wik, L. , Czarnewski, P. , Eldh, M. , Löf, L. , Ronquist, K. G. , Dubois, L. , Freyhult, E. , Gallant, C. J. , Oelrich, J. , Larsson, A. , Ronquist, G. , Villablanca, E. J. , Landegren, U. , Gabrielsson, S. , & Kamali‐Moghaddam, M. (2017). Tracing cellular origin of human exosomes using multiplex proximity extension assays. Molecular & Cellular Proteomics: MCP, 16(3), 502–511. 28111361 10.1074/mcp.M116.064725 PMC5341009

[jex270016-bib-0017] Lee, J. A. , Spidlen, J. , Boyce, K. , Cai, J. , Crosbie, N. , Dalphin, M. , Furlong, J. , Gasparetto, M. , Goldberg, M. , Goralczyk, E. M. , Hyun, B. , Jansen, K. , Kollmann, T. , Kong, M. , Leif, R. , McWeeney, S. , Moloshok, T. D. , Moore, W. , Nolan, G. , … Brinkman, R. R. (2008). MIFlowCyt: The minimum information about a Flow Cytometry Experiment. Cytometry. Part A: The Journal of the International Society for Analytical Cytology, 73(10), 926–930. 18752282 10.1002/cyto.a.20623 PMC2773297

[jex270016-bib-0018] Lee, K. , Fraser, K. , Ghaddar, B. , Yang, K. , Kim, E. , Balaj, L. , Chiocca, E. A. , Breakefield, X. O. , Lee, H. , & Weissleder, R. (2018). Multiplexed profiling of single extracellular vesicles. ACS Nano, 12(1), 494–503. 29286635 10.1021/acsnano.7b07060 PMC5898240

[jex270016-bib-0019] McNamara, R. P. , Zhou, Y. , Eason, A. B. , Landis, J. T. , Chambers, M. G. , Willcox, S. , Peterson, T. A. , Schouest, B. , Maness, N. J. , MacLean, A. G. , Costantini, L. M. , Griffith, J. D. , & Dittmer, D. P. (2022). Imaging of surface microdomains on individual extracellular vesicles in 3‐D. Journal of Extracellular Vesicles, 11(3), e12191. 35234354 10.1002/jev2.12191 PMC8888793

[jex270016-bib-0020] Pegtel, D. M. , & Gould, S. J. (2019). Exosomes. Annual Review of Biochemistry, 88, 487–514. 10.1146/annurev-biochem-013118-111902 31220978

[jex270016-bib-0021] Peinado, H. , Alečković, M. , Lavotshkin, S. , Matei, I. , Costa‐Silva, B. , Moreno‐Bueno, G. , Hergueta‐Redondo, M. , Williams, C. , García‐Santos, G. , Ghajar, C. , Nitadori‐Hoshino, A. , Hoffman, C. , Badal, K. , Garcia, B. A. , Callahan, M. K. , Yuan, J. , Martins, V. R. , Skog, J. , Kaplan, R. N. , … Lyden, D. (2012). Melanoma exosomes educate bone marrow progenitor cells toward a pro‐metastatic phenotype through MET. Nature Medicine, 18(6), 883–891. 10.1038/nm.2753 PMC3645291 22635005

[jex270016-bib-0022] Poliakov, A. , Spilman, M. , Dokland, T. , Amling, C. L. , & Mobley, J. A. (2009). Structural heterogeneity and protein composition of exosome‐like vesicles (prostasomes) in human semen. The Prostate, 69(2), 159–167. 18819103 10.1002/pros.20860

[jex270016-bib-0023] Santiago‐Dieppa, D. R. , Steinberg, J. , Gonda, D. , Cheung, V. J. , Carter, B. S. , & Chen, C. C. (2014). Extracellular vesicles as a platform for ‘liquid biopsy’ in glioblastoma patients. Expert Review of Molecular Diagnostics, 14(7), 819–825. 25136839 10.1586/14737159.2014.943193 PMC4436244

[jex270016-bib-0024] Singh, R. K. , Santos, M. F. , Herndon, C. , Gieler, B. A. , Lee, I. , Chen, J. , & Lorico, A. (2023). Detection by super‐resolution microscopy of viral proteins inside bloodborne extracellular vesicles. Extracellular Vesicles and Circulating Nucleic Acids, 4(4), 557–567. 10.20517/evcna.2023.46 PMC11648398 39697804

[jex270016-bib-0025] Théry, C. , Zitvogel, L. , & Amigorena, S. (2002). Exosomes: Composition, biogenesis and function. Nature Reviews Immunology, 2(8), 569–579. 10.1038/nri855 12154376

[jex270016-bib-0026] Tian, Y. , Gong, M. , Hu, Y. , Liu, H. , Zhang, W. , Zhang, M. , Hu, X. , Aubert, D. , Zhu, S. , Wu, L. , & Yan, X. (2020). Quality and efficiency assessment of six extracellular vesicle isolation methods by nano‐flow cytometry. Journal of Extracellular Vesicles, 9(1), 1697028. 31839906 10.1080/20013078.2019.1697028 PMC6896440

[jex270016-bib-0027] Tian, Y. , Ma, L. , Gong, M. , Su, G. , Zhu, S. , Zhang, W. , Wang, S. , Li, Z. , Chen, C. , Li, L. , Wu, L. , & Yan, X. (2018). Protein profiling and sizing of extracellular vesicles from colorectal cancer patients via flow cytometry. ACS Nano, 12(1), 671–680. 29300458 10.1021/acsnano.7b07782

[jex270016-bib-0028] van der Pol, E. , Coumans, F. A. , Grootemaat, A. E. , Gardiner, C. , Sargent, I. L. , Harrison, P. , Sturk, A. , van Leeuwen, T. G. , & Nieuwland, R. (2014). Particle size distribution of exosomes and microvesicles determined by transmission electron microscopy, flow cytometry, nanoparticle tracking analysis, and resistive pulse sensing. Journal of Thrombosis and Haemostasis: JTH, 12(7), 1182–1192. 24818656 10.1111/jth.12602

[jex270016-bib-0029] von Lersner, A. K. , Fernandes, F. , Ozawa, P. M. M. , Jackson, M. , Masureel, M. , Ho, H. , Lima, S. M. , Vagner, T. , Sung, B. H. , Wehbe, M. , Franze, K. , Pua, H. , Wilson, J. T. , Irish, J. M. , Weaver, A. M. , Di Vizio, D. , & Zijlstra, A. (2024). Multiparametric single‐vesicle flow cytometry resolves extracellular vesicle heterogeneity and reveals selective regulation of biogenesis and cargo distribution. ACS Nano, 18(15), 10464–10484. 38578701 10.1021/acsnano.3c11561 PMC11025123

[jex270016-bib-0030] Wei, P. , Wu, F. , Kang, B. , Sun, X. , Heskia, F. , Pachot, A. , Liang, J. , & Li, D. (2020). Plasma extracellular vesicles detected by Single Molecule array technology as a liquid biopsy for colorectal cancer. Journal of Extracellular Vesicles, 9(1), 1809765. 32944195 10.1080/20013078.2020.1809765 PMC7480466

[jex270016-bib-0031] Welsh, J. A. , Arkesteijn, G. J. A. , Bremer, M. , Cimorelli, M. , Dignat‐George, F. , Giebel, B. , Görgens, A. , Hendrix, A. , Kuiper, M. , Lacroix, R. , Lannigan, J. , van Leeuwen, T. G. , Lozano‐Andrés, E. , Rao, S. , Robert, S. , de Rond, L. , Tang, V. A. , Tertel, T. , Yan, X. , … van der Pol, E. (2023). A compendium of single extracellular vesicle flow cytometry. Journal of Extracellular Vesicles, 12(2), e12299. 36759917 10.1002/jev2.12299 PMC9911638

[jex270016-bib-0032] Yang, J. , Zhang, Y. , Gao, X. , Yuan, Y. , Zhao, J. , Zhou, S. , Wang, H. , Wang, L. , Xu, G. , Li, X. , Wang, P. , Zou, X. , Zhu, D. , Lv, Y. , & Zhang, S. (2021). Plasma‐derived exosomal ALIX as a novel biomarker for diagnosis and classification of pancreatic cancer. Frontiers in Oncology, 11, 628346. 34026608 10.3389/fonc.2021.628346 PMC8131866

[jex270016-bib-0033] Yoshioka, Y. , Kosaka, N. , Konishi, Y. , Ohta, H. , Okamoto, H. , Sonoda, H. , Nonaka, R. , Yamamoto, H. , Ishii, H. , Mori, M. , Furuta, K. , Nakajima, T. , Hayashi, H. , Sugisaki, H. , Higashimoto, H. , Kato, T. , Takeshita, F. , & Ochiya, T. (2014). Ultra‐sensitive liquid biopsy of circulating extracellular vesicles using ExoScreen. Nature Communications, 5(1), 1–8. 10.1038/ncomms4591 PMC3988821 24710016

[jex270016-bib-0034] You, Y. , Zhang, Z. , Sultana, N. , Ericsson, M. , Martens, Y. A. , Sun, M. , Kanekiyo, T. , Ikezu, S. , Shaffer, S. A. , & Ikezu, T. (2023). ATP1A3 as a target for isolating neuron‐specific extracellular vesicles from human brain and biofluids. Science Advances, 9(37), eadi3647. 37713494 10.1126/sciadv.adi3647 PMC10881047

[jex270016-bib-0035] Yuana, Y. , Koning, R. I. , Kuil, M. E. , Rensen, P. C. , Koster, A. J. , Bertina, R. M. , & Osanto, S. (2013). Cryo‐electron microscopy of extracellular vesicles in fresh plasma. Journal of Extracellular Vesicles, 2(1), 21494. 10.3402/jev.v2i0.21494 PMC3895263 24455109

[jex270016-bib-0036] Zhang, P. , Wu, X. , Gardashova, G. , Yang, Y. , Zhang, Y. , Xu, L. , & Zeng, Y. (2020). Molecular and functional extracellular vesicle analysis using nanopatterned microchips monitors tumor progression and metastasis. Science Translational Medicine, 12(547), eaaz2878. 32522804 10.1126/scitranslmed.aaz2878 PMC8024111

[jex270016-bib-0037] Zhang, P. , Zhou, X. , He, M. , Shang, Y. , Tetlow, A. L. , Godwin, A. K. , & Zeng, Y. (2019). Ultrasensitive detection of circulating exosomes with a 3D‐nanopatterned microfluidic chip. Nature Biomedical Engineering, 3(6), 438–451. 10.1038/s41551-019-0356-9 PMC6556143 31123323

[jex270016-bib-0038] Zhu, S. , Ma, L. , Wang, S. , Chen, C. , Zhang, W. , Yang, L. , Hang, W. , Nolan, J. P. , Wu, L. , & Yan, X. (2014). Light‐scattering detection below the level of single fluorescent molecules for high‐resolution characterization of functional nanoparticles. ACS Nano, 8(10), 10998–11006. 25300001 10.1021/nn505162u PMC4212780

