# Supplementary material for: Comparison of nanoimaging and nanoflow based detection of extracellular vesicles at a single particle resolution
Source: J Extracell Biol. 2024 Oct 16;3(10):e70016. doi: 10.1002/jex2.70016 (PMC11481688; doi:10.1002/jex2.70016)
Supplement: Supplementary file 1 — Supporting Information [file JEX2-3-e70016-s001.pdf]

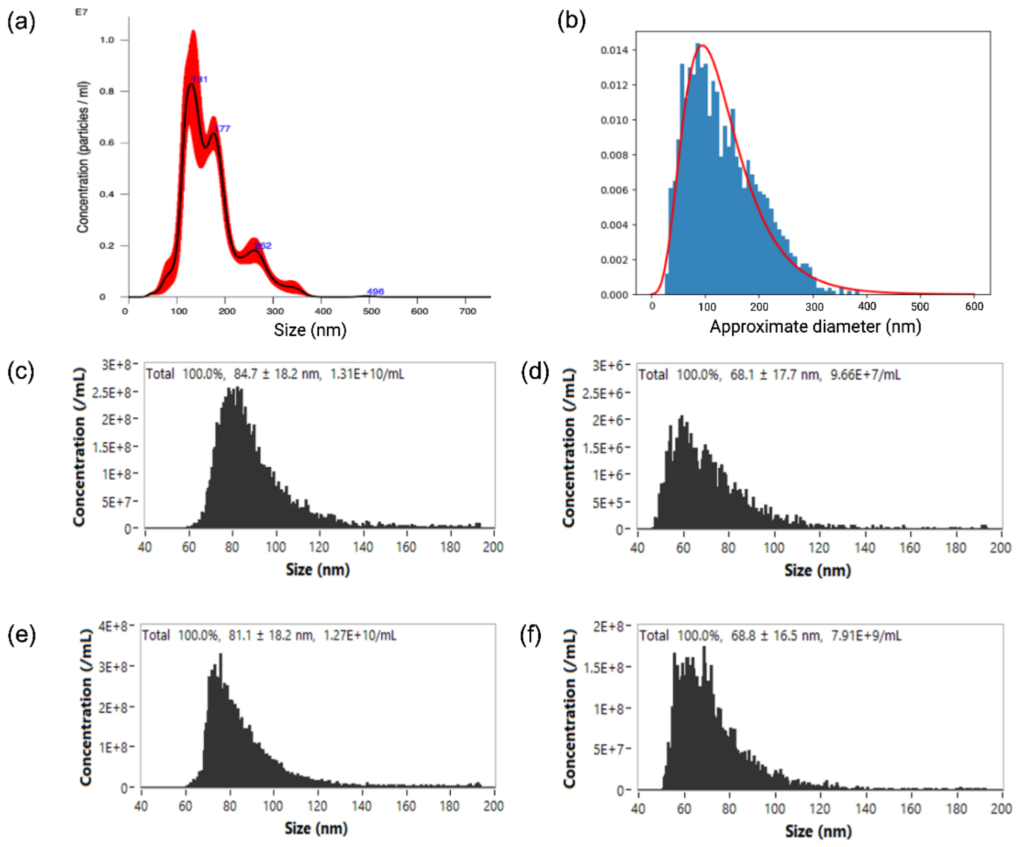

Figure S1. EVs were measured with NTA, Flow NanoAnalyzer and NanoImager.
(a) Size distribution from NTA. (b) Probability density distribution of 'approximate diameter' from NanoImager. (c) Size distribution from Flow NanoAnalyzer and concentration of EV particles considering dilution factors. EV samples were purified with (d) qEVsingle columns, Amicon filters for (e) once or (f) twice, and then assessed with Flow NanoAnalyzer.

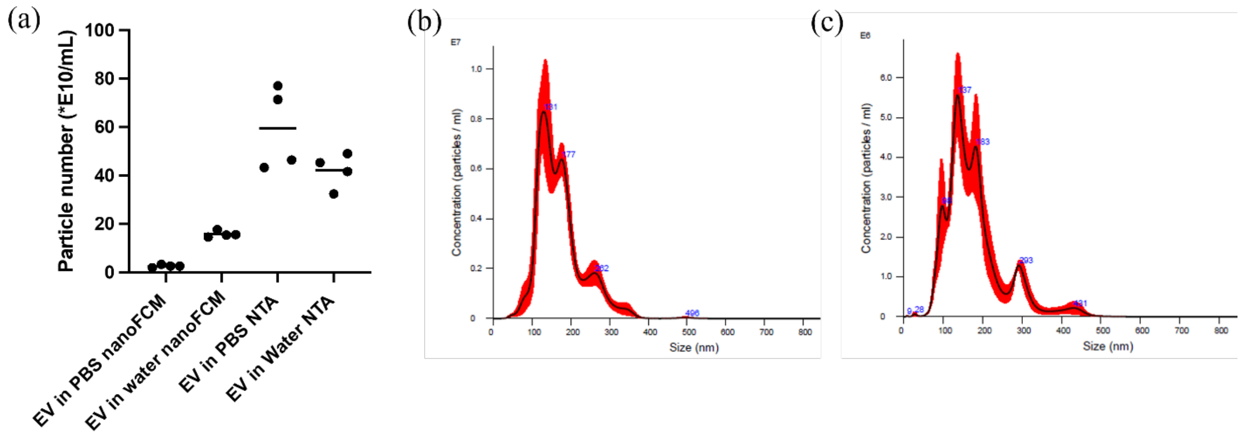

Figure S2. (a) The concentration of EVs measured by NanoFlow Analyzer (nanoFCM) and NTA. The concentration of EVs in PBS (mean value: 1.0*10^10/mL) or water (mean value: 1.6*10^11/mL) measured by Flow NanoAnalyzer, and in PBS (mean value: 5.8*10^11/mL) or water (mean value: 4.2*10^11/mL) measured by NTA. EV sizes were characterized with NTA in (b) PBS and (c) water. The mean value and standard error were 174.0 ± 6.3nm in PBS, 175.8nm ± 9.3nm in water.

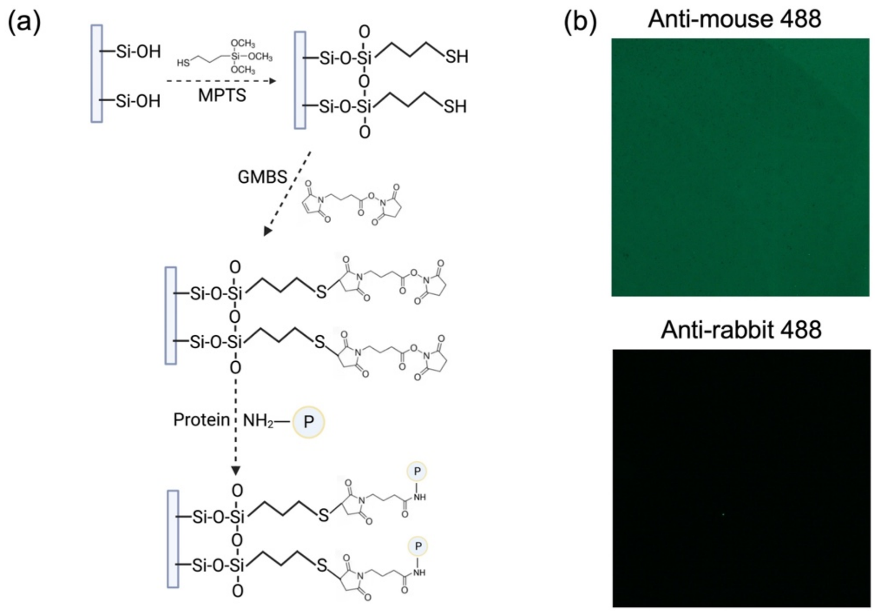

Figure S3. The scheme for chemical reactions on coverslips.

The functionality of MPTS and GMBS to bind with protein was confirmed with testing mouse-IgG. Alexa 488-conjugated anti-mouse IgG or Alexa 488-conjugated anti-rabbit IgG were added to target mouse-IgG.

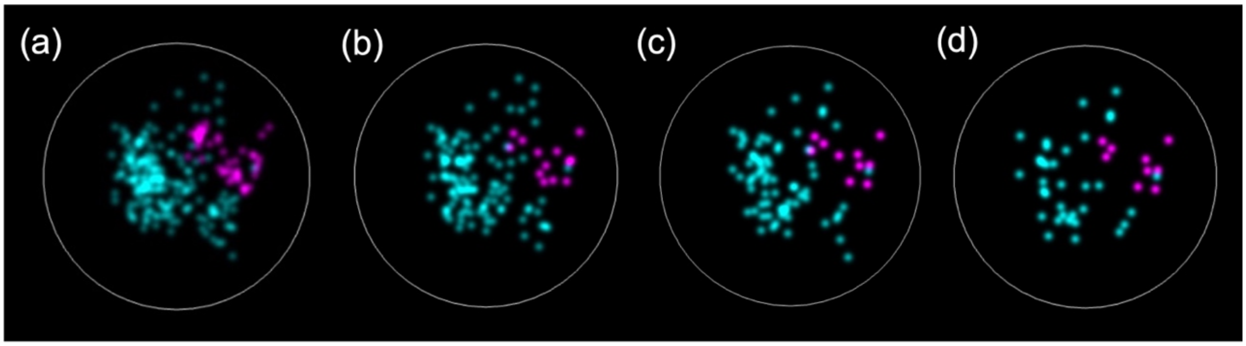

Figure S4. CODI settings for EV positivity analysis. (a) Without temporal grouping for localizations. (b) Maximum distance: 20 nm. (c) Maximum distance: 30 nm. (d) Maximum distance: 60 nm. With the above settings, the percentages for double positive EVs were: (a) 57.0%, (b) 56.4%, (c) 54%, and (d) 50.0%.

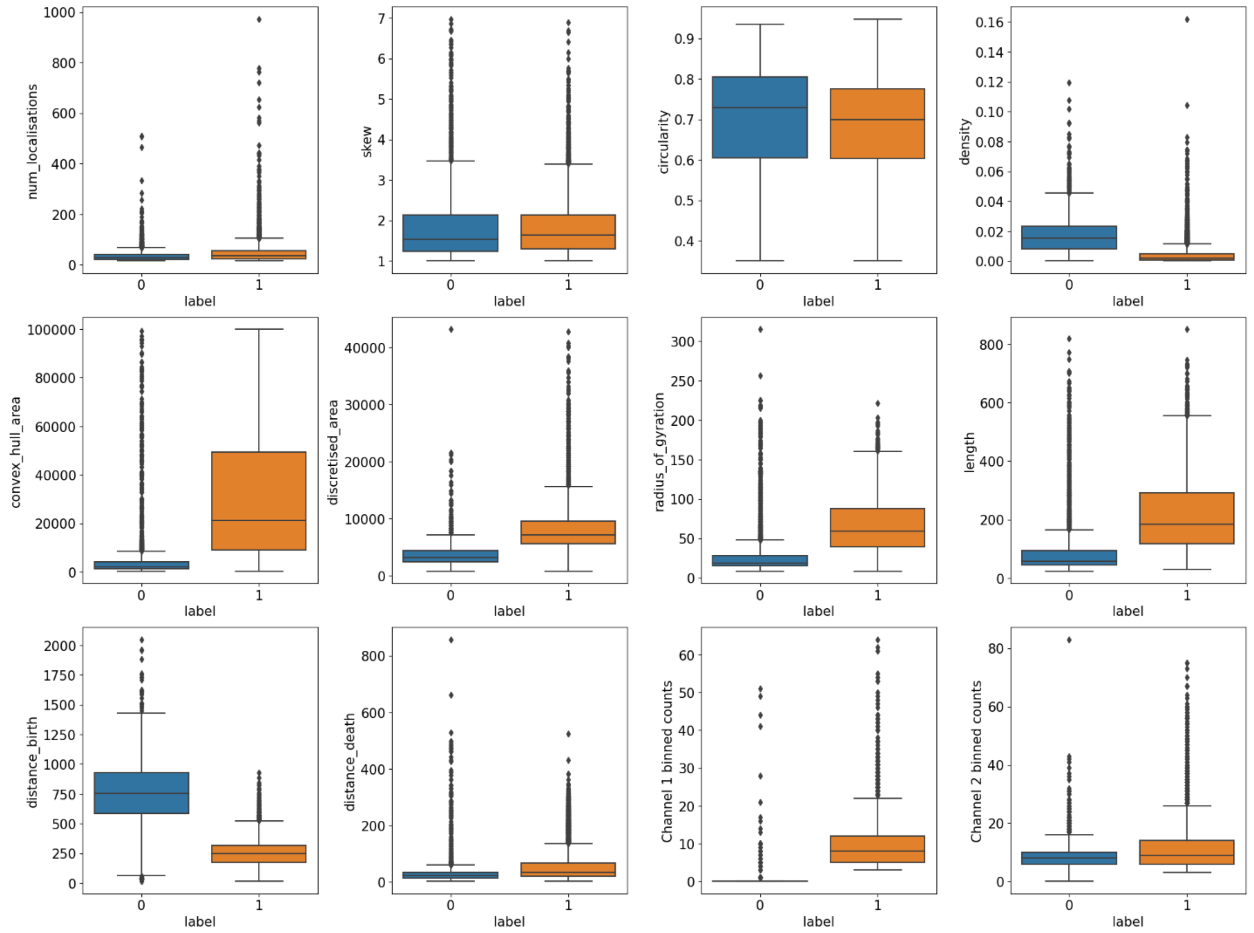

Figure S5. Analysis of CODI features. Different classes (label '0' as AB group and '1' as EV group) were compared across different features on images.

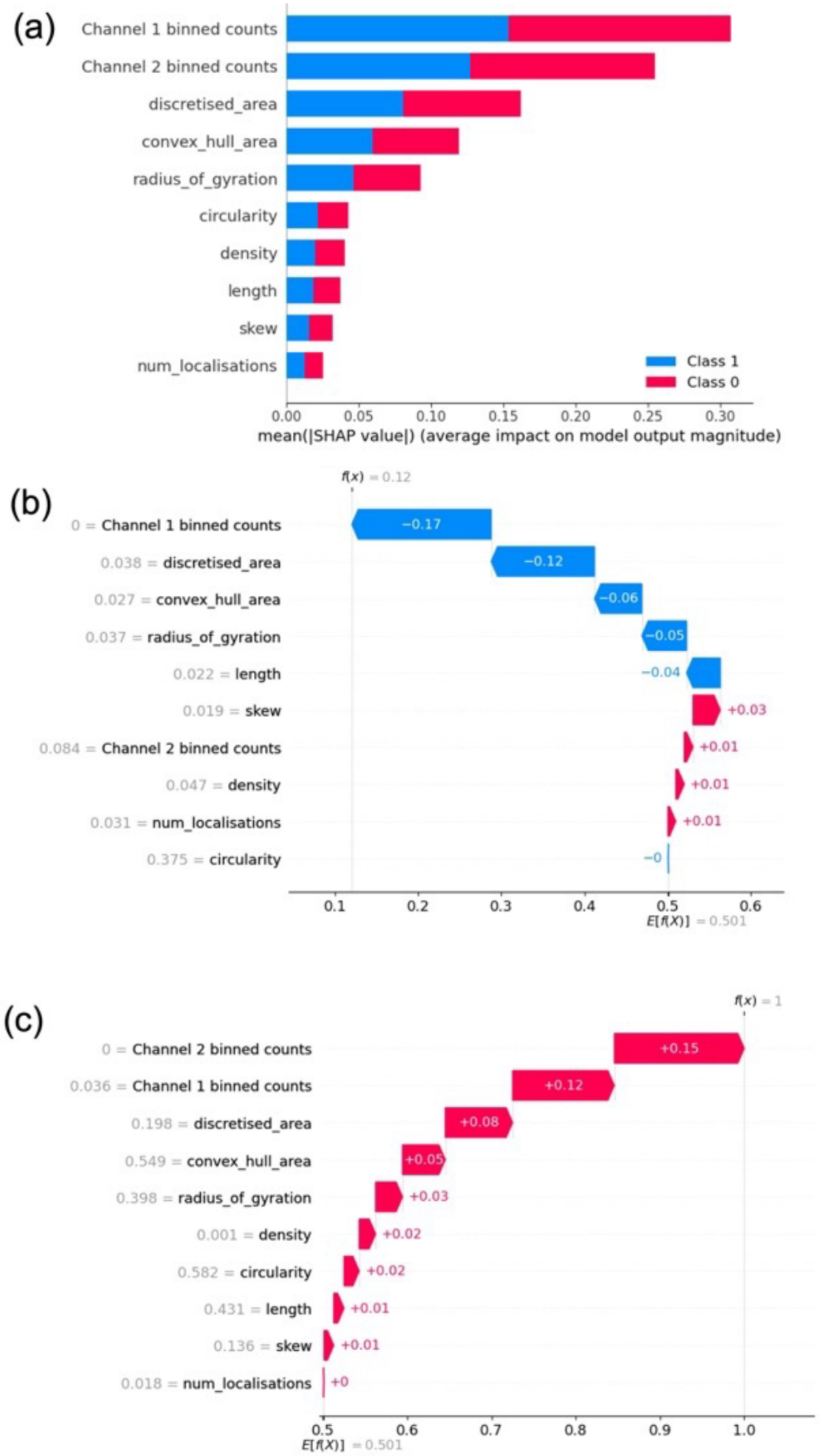

Figure S6. SHAP analysis of CODI features. (a) SHAP analysis was applied to visualize the importance of features. (b) For one double positive EV particle, it shows the contribution of each feature on predicting the particle as EV ('1'). (c) For false positive particles from antibody aggregates, it shows the contribution of each feature on predicting the particle as AB ('0').

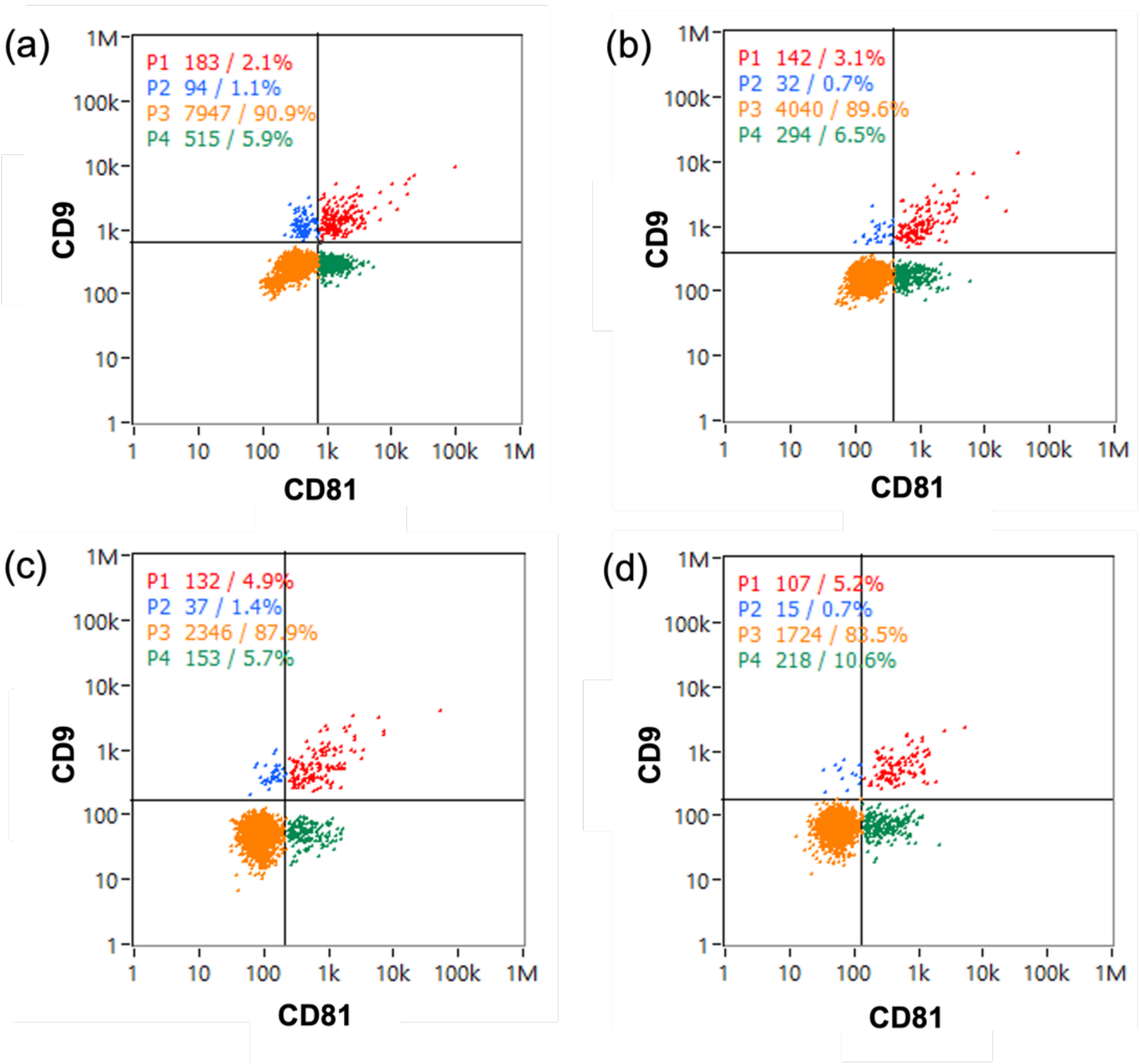

Figure S7. Scattered plots of fluorescence intensities of anti-CD81 and CD9 labeled EVs. (a) EVs were labeled with A488-anti CD9 and A647-anti CD81, and fluorescence signals were detected with Flow NanoAnalyzer after labeled EV samples were diluted with different dilution factors: (a) 50-fold, (b) 100-fold, (c) 200-fold, and (d) 400-fold.

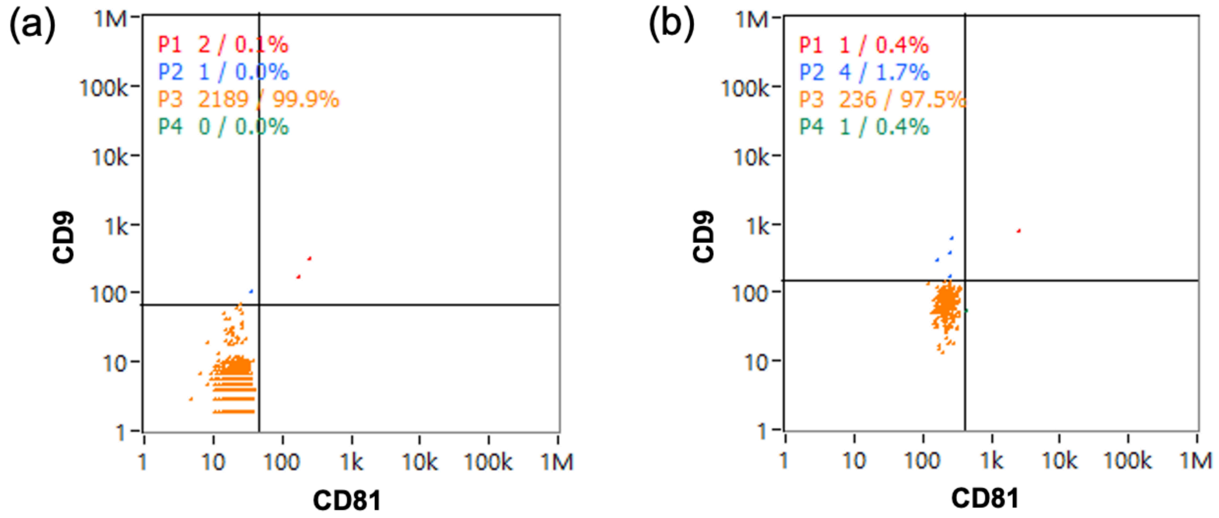

Figure S8. Scattered plot of CD81 and CD9 values. (a) EVs without labeling with antibodies and (b) mixture of A488-anti CD9 and A647-anti CD81 were characterized with Flow NanoAnalyzer as control samples.

Count	A647-CD81$^+$	A488-CD9$^+$	A647-CD81$^+$/ A488-CD9$^+$	A647-CD81$^+$	A488-CD9$^+$	A647-CD81$^+$/ A488-CD9$^+$
EV	627	779	2605	0.15632012	0.194215906	0.649464
	736	888	2376	0.184	0.222	0.594
	829	666	1958	0.240081089	0.19287576	0.5670432
	824	637	1897	0.245384157	0.189696248	0.5649196
	678	646	2161	0.194548063	0.185365854	0.6200861
	432	700	1370	0.172661871	0.279776179	0.547562
AB	5	439	2	0.011210762	0.984304933	0.0044843
	5	404	1	0.01219512	0.98536585	0.002439
	2	465	8	0.00421053	0.97894737	0.0168421
	6	430	4	0.01363636	0.97727273	0.0090909
	9	444	3	0.01973684	0.97368421	0.006579

Table S1. The counts for each phenotype, including A647-CD81$^+$, A488-CD9$^+$ and A647-CD81$^+$/A488-CD9$^+$ from dSTORM images were listed and their fractions were calculated for EV samples and control antibodies.

Sample-ML	Train accuracy	Test accuracy	Precision	Recall	F1	AUC-ROC
Data 1 -RF	1	0.99	0.98	0.98	0.98	0.999
Data 1 -LR	0.96	0.98	0.95	0.97	0.96	0.992
Data 1 -SVM	0.96	0.98	0.95	0.97	0.96	0.991
Data 2 -RF	1	0.99	0.98	0.96	0.97	0.998
Data 2 -LR	0.94	0.95	0.83	0.95	0.88	0.988
Data 2 -SVM	0.95	0.96	0.83	0.96	0.88	0.985
Data 3 -RF	1	0.985	0.98	0.97	0.98	0.998
Data 3 -LR	0.94	0.97	0.94	0.97	0.95	0.99
Data 3 -SVM	0.95	0.97	0.93	0.97	0.95	0.989
Data 4 -RF	1	0.99	0.99	0.97	0.98	0.999
Data 4 -LR	0.95	0.97	0.92	0.97	0.95	0.994
Data 4 -SVM	0.96	0.97	0.92	0.97	0.94	0.992

Table S2. The performance of different models on various datasets. ML: machine learning model, RF: random forest model, LR: logistic regression model, SVM: support vector machine model.
